# Variations in neuronal cytoskeletal integrity affect directed communication in distributed networks during inhibitory control

**DOI:** 10.1038/s42003-025-07974-4

**Published:** 2025-03-29

**Authors:** Julia Elmers, Moritz Mückschel, Katja Akgün, Tjalf Ziemssen, Christian Beste

**Affiliations:** 1 Cognitive Neurophysiology, Department of Child and Adolescent Psychiatry, Faculty of Medicine, TU Dresden, Germany; 2 Center of Clinical Neuroscience, Department of Neurology, University Hospital Carl Gustav Carus, TU Dresden, Germany; 3 German Center for Child and Adolescent Health (DZKJ), Partner Site Leipzig/Dresden, Dresden, Germany

**Keywords:** Cognitive control, Sensorimotor processing, Human behaviour

## Abstract

To ensure goal-directed behavior in daily life, the use of inhibitory control is of great importance. The aim of this study is to shed light on the underlying neuronal mechanisms of inhibitory control and the relevance of cytoarchitectonic integrity in it. We combine sophisticated EEG analysis techniques assessing directed communication between brain structures with measurements of neurofilaments as an index of cytoarchitectonic integrity. We show that an extensive theta band activity related neural network with fronto-temporal, parietal, and occipital brain regions is active during response inhibition. Importantly, cytoarchitectonic integrity as measured using neurofilaments modulates nonlinear directional connectivity, particularly when complex reconfiguration of perceptual and action mapping is required. The study thus shows an inter-relation between different levels of biological functioning—the level of cytoarchitectonic integrity and neurophysiological directed communication—for inhibitory control and emphasizes the role of nonlinear brain connectivity in cognitive control.

## Abstract (web summary)

Cytoskeletal integrity affects neurophysiological directed communication important for goal-directed action

## Introduction

In daily life, individuals are often confronted with situations where the automatic or prepotent response is not necessarily the most appropriate response. In that case, inhibitory control is needed to withhold inappropriate responses (inhibition of actions) or shield from the distraction of irrelevant stimuli (inhibition of interferences) to allow goal-directed behavior^1,2^. Previous research suggests that the inhibition of actions and the inhibition of interferences—two core aspects of inhibitory control—are interdepended^3,4^ and require the integration of multiple stimuli and responses features with use of cognitive control^5^.

Achieving such inhibitory/cognitive control, however, depends on the brain’s ability to process information both locally and globally across distant regions^6–8^. To ensure global processing, communication between distant brain regions (i.e., in the fronto-parietal network) is necessary. It is theorized that distant brain regions communicate through synchronized oscillatory activity, particularly slow wave theta oscillations (4–7 Hz), which facilitate brain-wide coordination^9,10^. Importantly, theta band activity is also crucial for cognitive control processes^11^ and multiple lines of evidence show theta band activity modulations before and during response inhibition processes^12–14^, as well as during conflict monitoring^15–18^. Several brain regions contribute to inhibitory control. Initially, it has been stated that the right inferior frontal gyrus (IFG) is specifically dedicated to inhibitory processes^1,2,19^. This modular view was later revised, suggesting that the right IFG, along with the pre-supplementary motor areas, is crucial for inhibitory control^1^. Moreover, a growing body of research suggests that inhibitory control is not confined to a single brain region^20^, because inhibitory tasks also involve other cognitive processes depending on a distributed network^20^. It is well-known that network-dependent processes subserve executive functions^21,22^ and it is suggested that the interdependency of different networks like the default mode network, the salience network, and the central executive network is crucial for cognitive control^23^. Evidence thereby speaks for the involvement of a fronto-parietal network during inhibitory control. In more detail, we suggest the following regions to be involved in (theta-modulated) automatic and cognitive control of response inhibition. The temporoparietal junction (TPJ), for example, is crucial for encoding relevant stimuli to resolve conflicting contexts^24^ and is associated with modulations in “response selection codes”^4,25^. Further, the superior parietal lobule, including the precuneus, plays an essential role in response inhibition^3,26^. Finally, the insula and the anterior cingulate cortex, both part of the salience network, are involved in successful response inhibition, conflict monitoring, and interference control^4,27–30^.

While an extended body of literature shows multiple brain regions to be involved in response inhibition, it is still not understood how information is exchanged between these regions, likely constituting a network. This is all the more the case when considering oscillatory activity, which is essential to enable distant communication between brain structures. Outlining the directed exchange of information between brain regions is one goal of the current study. The relevance of such questions on information transfer is underlined by the recent spatiotemporal neuroscience approach^31,32^.

The second goal of this study is to provide insights into the biological determinants of directed communication between brain structures during inhibitory control. One important determinant is the integrity of neural structure impacting functional connectivity^33^. Factors determining cytoskeletal integrity are neurofilaments (NFs), which form the neuronal cytoskeleton together with other cytoskeletal proteins. NFs are hetero-polymers composed of a light (NFL), an intermediate (NFM), and a heavy (NFH) chain together with α-internexin^34,35^. Disruption of the neural integrity as indexed by NFs, and NFL in particular, affects cognitive functioning^36–40^, possibly because network-like functional organization in the brain is affected^41^. Thereby, especially the integrity of long-distance white matter tracts of distributed networks (e.g., fronto-parietal networks) is particularly crucial for complex behavior^42–45^. In the current study, we examine how variations in NFL affect directed communications between the brain involved in the inhibition of actions. For this, we focus on theta band activity and use blood serum samples as NFL leaks into the cerebrospinal fluid and subsequently into the bloodstream, where it can be detected and measured in serum samples (sNFL) correlating with CSF release^46–48^.

When considering directed communication or the transfer of information between brain regions, it is important to distinguish between directed linear and nonlinear communication regimens. While much research has focused on linear interactions between these areas, emerging evidence suggests an important role of nonlinear connections between brain regions^49–51^. Especially when it comes to processes requiring the integration of perception and action in feedforward and feedback loops^52,53^, linear and nonlinear dynamic plays a role^54–56^. Nonlinear functional connectivity patterns are constrained by the anatomical pathways that form the “backbone” of these interactions. Disruption of these pathways, even in small ways, can lead to large-scale changes in the functional connectivity profile^57,58^. To conduct a detailed examination of directed linear and nonlinear connectivity between brain regions, we applied nonlinear Causal Relationship Estimation by Artificial Neural Network (nCREANN)^59–61^. This method allowed us to illuminate the specific directed linear and nonlinear connectivity profile underlying response inhibition. We expect that directed connectivities between brain regions associated with theta band activity during response inhibition are modulated by the sNFL levels. To experimentally vary demands on response inhibition processes and thereby on the likely relevance of directed communication between involved brain structures, we task demands were varied through more automated or controlled processing of stimulus–response associations^4^. Taken together, the study aims to test a possible inter-relation between different levels of biological functioning—the level of cytoarchitectonic integrity and neurophysiological directed communication.

## Results

The Simon Nogo Task can be seen as a hybrid of a Simon Task and a Go/Nogo Task, whereby participants had to withhold a motor reaction in NOGO trials (indicated by bold, italic letters). Further, trials differed in compatibility, namely compatible trials (letter “A” on the left side) and incompatible trials (letter “A” on the right side). For clarity, compatible trials mirror an automated processing style, as the response selection (i.e., key press) depends only on the spatial location of the stimulus. In incompatible trials, on the other hand, cognitive conflicts increase as the ‘automatic unconditional’ response tendency based on stimulus laterality is opposed by a ‘controlled conditional’ response selection (i.e., based on task instructions). For a detailed description, see the “Methods” section.

### Behavioral results

**Fig. 1:**
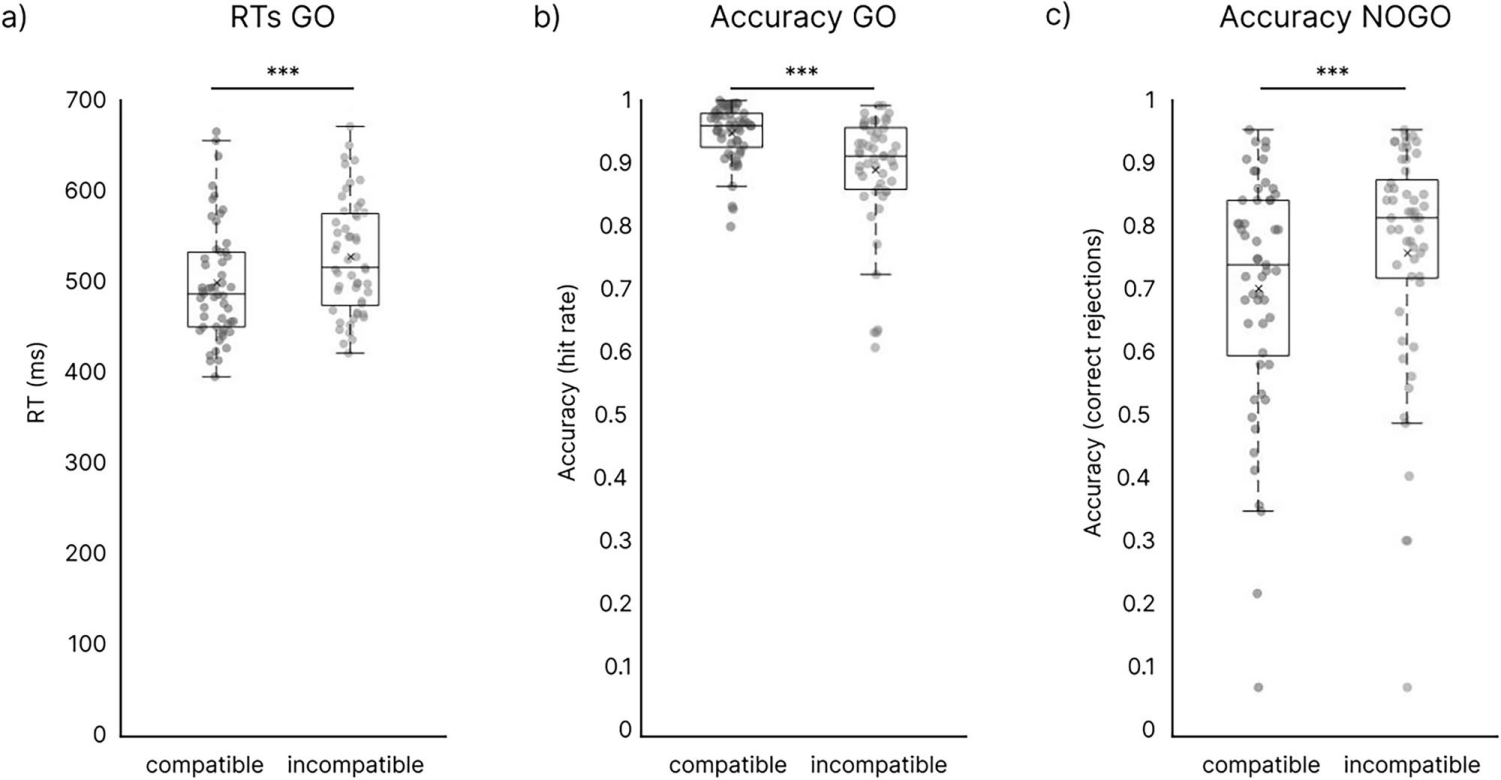
Boxplots of behavioral results from the Simon Nogo Task. Shown are **a** mean reaction times (RTs) and **b** mean hit rates for the GO trials, as well as **c** mean correct rejection rates for the NOGO trials of *N* = 53 participants. ***p < 0.001. Center line = median; × = mean; box limits = upper and lower quartiles, whiskers = minimum and maximum within 1.5x interquartile range, circles = individual points.

The results of behavioral data are shown in Fig. 1. A paired-sample *t*-test revealed faster reaction times (RTs) in compatible (495 ± 64 ms) vs. incompatible (524 ± 63 ms, (*t* (53) = -8.418, *p* < 0.001, *d* = 1.156) GO trials. Regarding accuracy (i.e., hit rate in GO trials and correct rejection rate in NOGO trials), the repeated measures ANOVA revealed a main effect of *condition* (*F*(1,52) = 95.975, *p* < 0.001, partial *η*^2^ = 0.649) with higher accuracy in GO trials (92.2 ± 0.8%) compared to NOGO trials (72.0 ± 2.5%). No main effect of *compatibility* was observed (*F*(1,52) = 0.055, *p* = 0.816, partial *η*^2^ = 0.001). However, the interaction between factors *condition* and *compatibility* was significant (F(1,52) = 44.272, *p* < 0.001, partial *η*^2^ = 0.46). A post-hoc paired-sample t-test was conducted for GO and NOGO trials separately. To account for multiple comparisons, a Bonferroni correction was applied, adjusting the significance level to α = 0.025 for each test. In GO trials, we found higher accuracy in compatible than in incompatible trials (compatible: 95.04 ± 4.44%; incompatible: 89.41 ± 8.41%; *t*(52) = 5.893, *p* < 0.001, *d* = 0.809). Opposed to this, in NOGO trials, the accuracy in the compatible trials was lower than in the incompatible trials (compatible: 69.30 ± 19.09%; incompatible: 74.62 ± 18.91%; *t*(52) = -4.541, *p* < 0.001, *d* = 0.624). This pattern was found repeatedly in previous studies^4,24,26^ and shows that response inhibition is more demanding (error-prone) in the compatible than in the incompatible condition.

### Neurophysiological results

#### Oscillatory signatures of inhibitory control

**Fig. 2:**
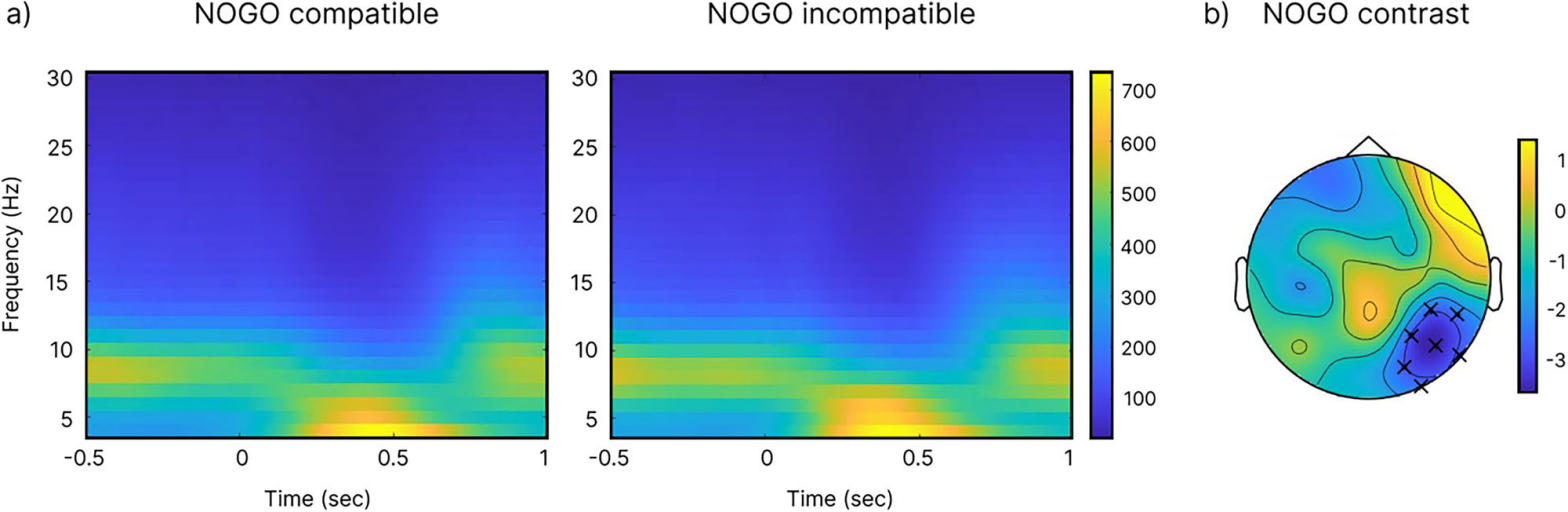
Results of the time-frequency analysis. **a** displays time-frequency plots of power at electrodes Cz and FCz for NOGO compatible and incompatible trials. **b** shows the result of the cluster-based permutation test between conditions (i.e., NOGO compatible—NOGO incompatible) for the time window of 300 to 600 ms in the theta frequency band (single image shows the results averaged over time). Significant differences (*p*  < 0.05) are represented by a ‘×’.

An effect between the compatible and incompatible NOGO trials was seen in the theta frequency band, between 300 and 600 ms after stimulus initiation, according to the cluster-based permutation test. Theta power was greater in NOGO incompatible trials, especially at right posterior electrodes (Fig. 2).

Dynamic imaging of coherent sources (DICS) beamforming analyses with subsequent voxel clustering using the Density-Based Spatial Clustering of Applications with Noise (DBSCAN) algorithm were conducted for both NOGO conditions after assessing the significant differences at the sensor level were determined (for a detailed description, see Methods section). The obtained regions, hereafter activity clusters (ACs), and their corresponding Brodmann areas are displayed in Table 1.

**Table 1:** Activity clusters (ACs) for both NOGO conditions

AC		Brain regions	Brodmann areas
1	Left fronto-temporal cluster	**Temporal pole** **Middle and superior temporal gyrus** **Frontal inferior** **Insula** **Postcentral gyrus** Rolandic operculum	BA 38 BA 21 and BA 22/41/42 BA 44/45/47 BA 13/16 BA 1/2/3 BA 43
2	Bilateral occipital cluster	**Middle (and superior) occipital gyrus** **Cuneus** **Calcarine sulcus** Lingual gyrus^b^	BA 18 and BA 19 BA 17/19 BA 17 BA 19
3	Right temporo-parietal cluster	**Supramarginal gyrus** **Rolandic operculum** Superior temporal gyrus Postcentral gyrus^a^	BA 40 BA 43 BA 22/41/42 BA 1/2/3
4	Left parietal cluster	**Inferior parietal gyrus** **Postcentral gyrus** Supramarginal gyrus^b^ Rolandic operculum^b^ Superior temporal gyrus^b^	BA 39/40 BA 1/2/3 BA 40 BA 43 BA 22/41/42

*Note*. Bold regions = more than 5 voxels.

^a^Only found in NOGO compatible trials.

^b^Only found in NOGO incompatible trials.

#### Linear and nonlinear directed connectivity

**Fig. 3:**
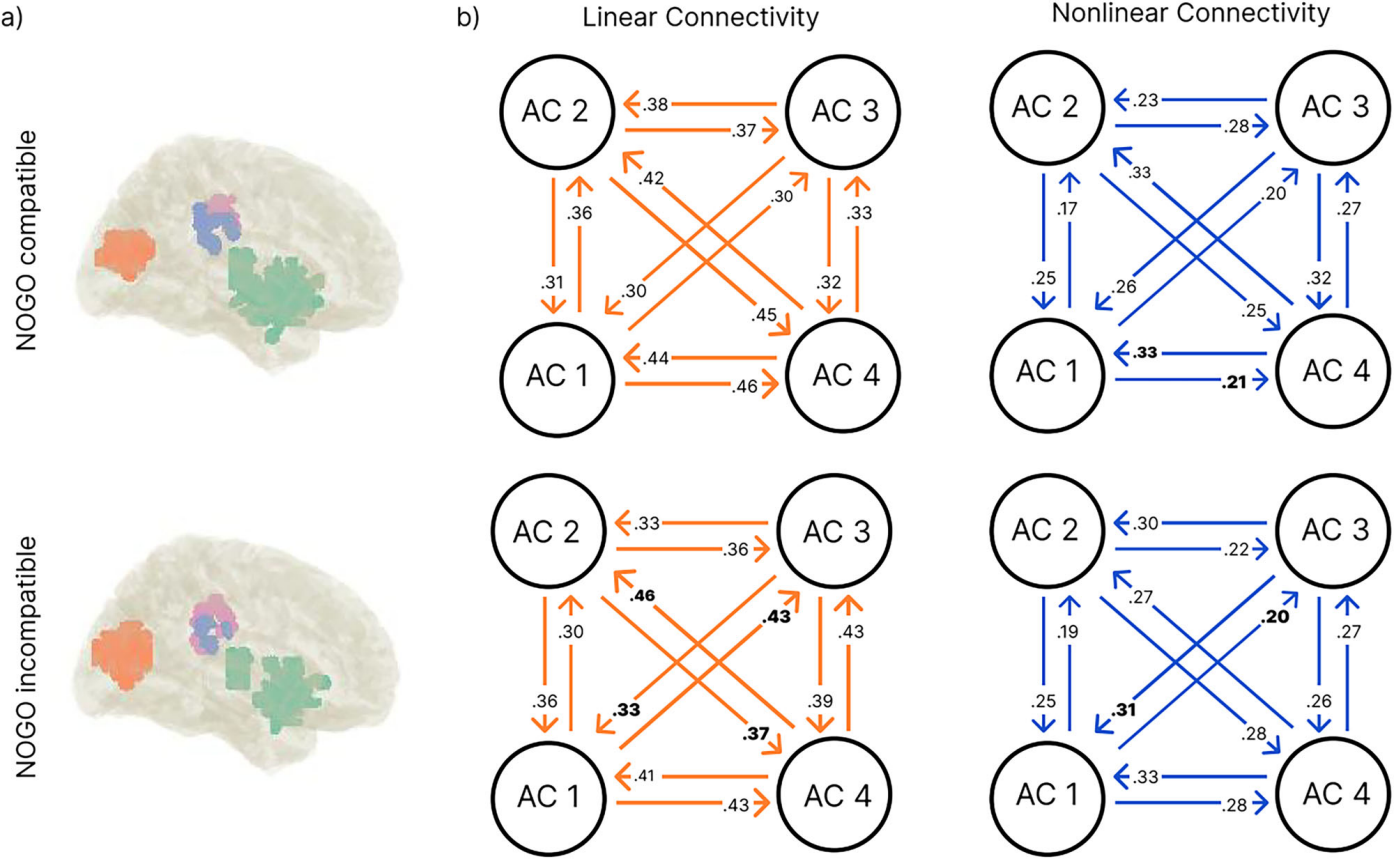
Results of the nCREANN algorithm. Data shown are average values across all subjects (i.e., on a group level). Activity clusters (ACs) in both conditions are shown in (**a**) for compatible and incompatible NOGO conditions with AC 1 (green) = left fronto-temporal cluster, AC 2 (orange) = bilateral occipital cluster, AC 3 (purple) = right temporoparietal cluster, AC 4 (pink) = left parietal cluster. **b** shows single non-self connectivities for each condition for the linear (orange) and nonlinear (blue) connectivity analyses. Note that all connections reflect meaningful connections (i.e., directed information transfer) since surrogate data testing with 100 permutations was applied. Bold numbers indicate a significant difference (*p* <  0.05) between the directed connections from and to an AC.

The nCREANN algorithm revealed connectivity patterns in the reported network, including the left fronto-temporal, the bilateral occipital, the right temporoparietal, and the left parietal activity cluster. In Fig. 3, the linear and nonlinear connectivity patterns are displayed for the averaged values across all subjects for compatible and incompatible NOGO trials. There were almost no significant differences between connectivities from one AC to another, meaning that all ACs showed similar incoming and outgoing connectivity values. In the linear network of incompatible NOGO trials (Fig. 3), effective connectivity was stronger from AC 1 to AC 3, and from AC 4 to AC 2 than vice versa. In the nonlinear network, directed connectivity was stronger from AC 4 to AC 1 than the other way around in compatible NOGO trials, and stronger from AC 3 to AC 1 than contrariwise in incompatible NOGO trials (all *p* < 0.05 using dependent *t*-tests).

When comparing the averaged network connectivities between compatible and incompatible NOGO trials, no significant difference was observed (linear: *t*(53) = -0.397, *p* = 0.693; nonlinear: *t*(53) = −0.550, *p* = 0.585).

### The role of sNFL for directed connectivity

The mean sNFL level was 6.55 ( ± 3.19) pg/ml, which is a heightened value compared to other studies with healthy controls^41^ but still in a non-pathological range^62–65^.

Regarding behavioral data, the correlational analysis revealed one moderate negative correlation of sNFL with the correct rejection rate in incompatible NOGO trials (*r* = −0.297, *p* = 0.043). Other correlations between sNFL and behavioral RT or accuracy measures were not significant (all *p* > 0.095).

**Fig. 4:**
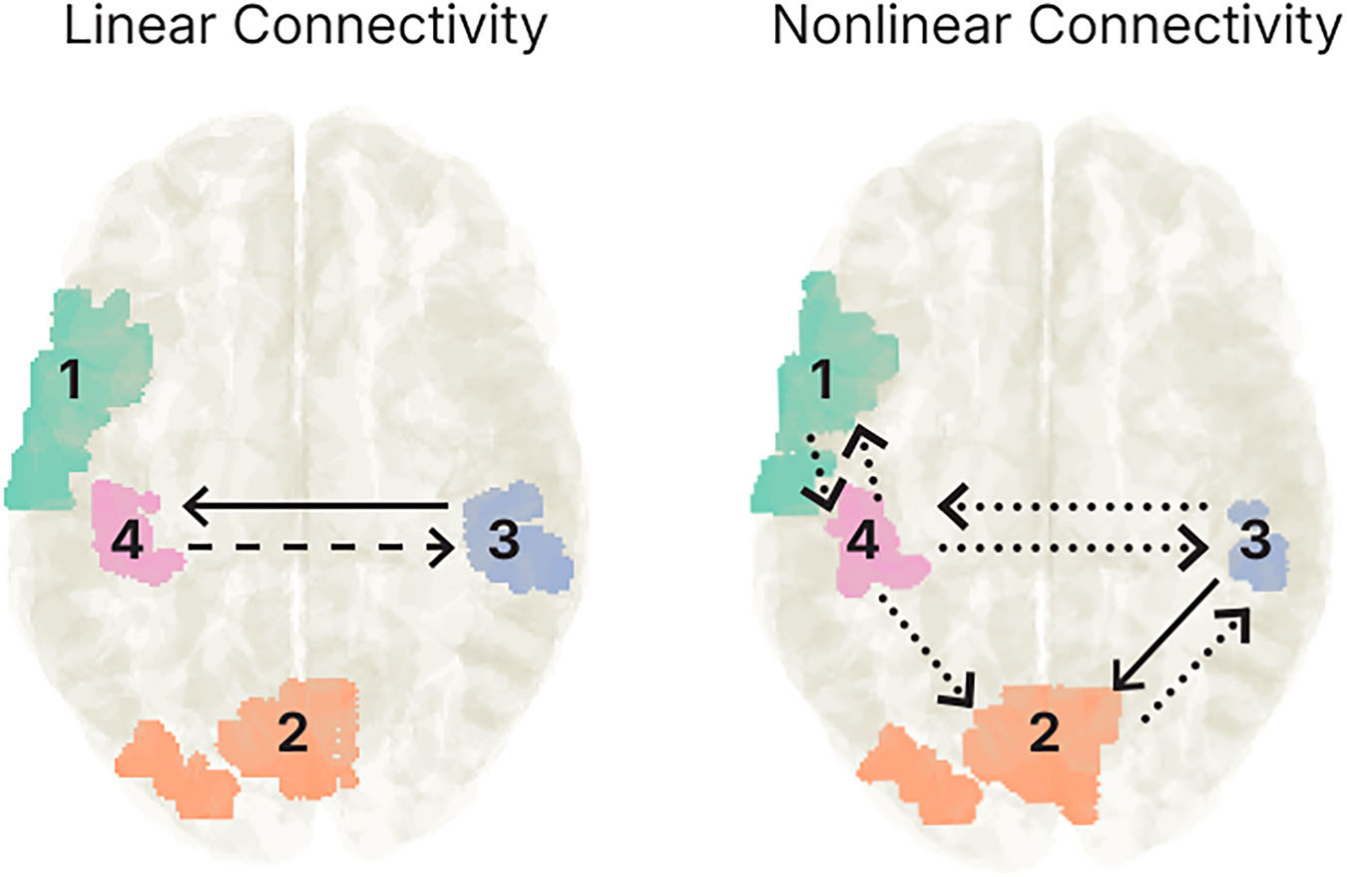
Correlations between effective connectivity values and sNFL. Connections between ACs that significantly correlated with sNFL values when considering *p*-values corrected for multiple comparisons. Dotted lines show significant correlations, which were only found in NOGO incompatible condition. The dashed line indicates a significant correlation that was only found in NOGO compatible conditions. The solid line indicates that significant correlations were found in both conditions.

Correlational analysis with connectivity measures showed significant moderate to strong correlations between the averaged network connectivities with sNFL values for both linear and nonlinear networks. For linear networks, we found correlations for compatible (*r* = 0.468, *p* < 0.001) and incompatible (*r* = 0.310, *p* = 0.023) NOGO trials with sNFL. For nonlinear networks, a descriptively greater correlation of sNFL with incompatible (*r* = 0.468, *p* < 0.001) than with compatible (*r* = 0.282, *p* = 0.039) NOGO trials was evident. We further looked at the relationship of sNFL values with single connectivities in the network (e.g., connectivity from one AC to another). The results are shown in Table 2. Especially for nonlinear connections to and from AC 4 (left parietal cluster), we found stable correlations with sNFL values (i.e., survived FDR correction) in incompatible NOGO trials. Significant correlations are also visualized in Fig. 4.

**Table 2:** Results of correlational analysis between sNFL values and single connectivities derived from nCREANN

Linear						
	NOGO compatible			NOGO incompatible		
AC → AC	rho	*p*	Corrected p*	rho	*p*	Corrected p*
1 → 1	0.059	0.670	0.670	0.078	0.575	0.913
2 → 1	0.176	0.203	0.372	0.012	0.931	0.931
3 → 1	0.079	00.572	0.653	0.067	0.630	0.913
4 → 1	0.213	0.121	0.343	0.328	0.**016**	0.071
1 → 2	0.218	0.113	0.343	0.154	0.267	0.535
2 → 2	0.084	0.546	0.653	−0.050	0.721	0.913
3 → 2	0.292	0.**032**	0.171	0.321	0.**018**	0.071
4 → 2	0.155	0.262	0.407	0.140	0.314	0.557
1 → 3	0.150	0.280	0.407	0.035	0.799	0.913
2 → 3	0.209	0.129	0.343	0.219	0.112	0.298
3 → 3	0.174	0.209	0.372	−0.041	0.767	0.913
4 → 3	0.372	0.**006**	0.**045**	0.254	0.063	0.203
1 → 4	0.135	0.330	0.440	0.324	0.**017**	0.071
2 → 4	0.184	0.184	0.372	0.204	0.140	0.319
3 → 4	0.387	0.**004**	0.**045**	0.397	0.**003**	0.**048**
4 → 4	0.070	0.616	0.657	0.019	0.892	0.931

Nonlinear						
	NOGO compatible			NOGO incompatible		
AC → AC	rho	*p*	corrected p*	rho	*p*	corrected p*
1 → 1	0.244	0.075	0.336	0.228	0.098	0.127
2 → 1	0.112	0.422	0.614	0.087	0.530	0.530
3 → 1	−0.027	0.847	0.903	0.224	0.103	0.127
4 → 1	0.057	0.683	0.781	0.339	0.**012**	0.**024**
1 → 2	−0.093	0.503	0.670	0.232	0.092	0.127
2 → 2	0.266	0.052	0.336	0.200	0.148	0.169
3 → 2	0.456	0.**001**	0.**009**	0.515	0.**000**	0.**001**
4 → 2	0.214	0.120	0.336	0.502	0.**000**	0.**001**
1 → 3	−0.068	0.623	0.767	0.238	0.084	0.127
2 → 3	0.192	0.164	0.336	0.425	0.**001**	0.**005**
3 → 3	0.199	0.149	0.336	0.183	0.186	0.198
4 → 3	0.182	0.189	0.336	0.348	0.**010**	0.**023**
1 → 4	0.012	0.930	0.930	0.371	0.**006**	0.**015**
2 → 4	0.124	0.372	0.595	0.298	0.**029**	0.051
3 → 4	0.183	0.185	0.336	0.563	0.**000**	0.**000**
4 → 4	0.188	0.173	0.336	0.372	0.**006**	0.**015**

*Note*. Significant *p*-values are printed in bold. *p-values were adjusted for each condition with the false-discovery rate (FDR) method. *AC* activity cluster.

## Discussion

The goal of the current study was to delineate the role of NFs as an index for neuronal cytoarchitectonic integrity for the directed communication between brain structures during response inhibition. As a parameter to account for neuronal cytoarchitectonic integrity, we used sNFL levels. The study thus shows an inter-relation between different levels of biological functioning—that is, the level of cytoarchitectonic integrity and neurophysiological directed communication during cognitive control. Within a distributed fronto-parietal network of theta band activity during response inhibition, especially nonlinear directed communication in this network was associated with the neuronal cytoarchitectonic integrity.

The behavioral data line up with previous findings showing that response inhibition is more demanding (error-prone) in compatible vs. incompatible NOGO trials of the applied Simon Nogo Task^3,4^. The reason for this is that during compatible trials the mapping of the stimulus input to the motor output likely operates via a so-called ‘direct route’^66–69^ and an automatic tendency to respond towards the stimulus location. In incompatible trials, a conflict between ‘unconditionally automatic’ response tendencies, indicating the stimulus laterality, and a ‘controlled conditional selection’ of stimulus features, indicating the response button, emerge^66–69^. This conflict complicates response selection processes, makes responding less automatic, and, therefore, easier to inhibit. Similar conclusions are also evident when using accounts on how perception and motor processes become integrated during response selection^5^.

While several frequency bands and their interplay have been associated with inhibitory control, notably alpha, beta, gamma, and theta^12,70–73^, especially theta band activity is linked to the reconfiguration of perception-action associations, particularly when tasks are demanding^74,75^ and recent evidence suggest that the reconfiguration of perception-action associations is at the core of inhibitory control processes^76,77^. In the current study, we therefore focus on theta band activity, which was modulated by the experimental variations of a more automatic (demanding) and controlled (less demanding) experimental condition. Theta band activity was elevated during incompatible NOGO trials, where response selection—including the decision not to inhibit a response—requires a complex reorganization of how perceptual information is mapped onto motor output^78^.

On a functional neuroanatomical level, we found a brain-wide network of four ACs implicated in the Simon Nogo Task, including fronto-temporal, parieta,l and occipital brain regions. First, a left-sided fronto-temporal AC encompassing the temporal pole, the middle and superior temporal gyrus, the insular cortex, and the IFG (i.e., AC 1) was evident. These regions have all not only previously been associated with the widespread ventral attentional network^79^, but are also suggested to support various processes of cognitive control. Specifically, the temporal pole is considered a cortical convergence zone and cross-modal hub^80–82^. It is also suggested to filter coherent concepts from different input modalities^83^ and to play a role in action planning and selection^79^. Further, the middle temporal gyrus is important for the process of stimulus–response mapping and action selection^84–86^. The insula is not only involved in several processes regarding interference control, response inhibition, perception-action integration, and action planning/selection^8,27,29,30,79,87^, but also important for the estimation of demand of cognitive control^88^. Lastly, the IFG was shown to be active during the detection and resolving of interference^27,30,87,89,90^, and inhibitory motor control^29,79,91^. Moreover, the IFG is also part of the fronto-parietal network, which is crucial for the modulation of cognitive control demands^92^. Second, there were bilateral activity modulations in the middle (and superior) occipital gyrus (MOG) and the cuneus (i.e., AC 2). Previous research showed that activation in the MOG and the cuneus was associated with endogenous attentional resource allocation (i.e., top-down attentional control)^93^ and with cognitive interference control^87^. Further, the (pre-)cuneus is suggested to modulate theta band activity during inhibitory control processes^26^. For AC 3, especially the activation in the right supramarginal gyrus is of interest as it is—like the IFG—part of the fronto-parietal network of cognitive control, especially involved in inhibitory control^94^ and flexible response selection^95,96^. Lastly, AC 4 was mainly formed from voxels in the left inferior parietal gyrus, also often referred to as the temporoparietal junction (TPJ). The TPJ is also involved in inhibitory control as it updates internal representations with incoming sensory information^25,97^ for subsequent action plans^3,4,24,97,98^. However, the found network did not include the cingulate cortex, as indicated by ref. ^4^, using the very same task. This might be due to differences in the methodology as^4^ used another source localization method on their neurophysiological data and found the cingulate cortex to be the source of the difference in the N2 event-related potential, which likely reflects modulatory effects of sensorimotor transformation processes on response inhibition. Taken together, all found ACs are strongly associated with processes involved in the Simon Nogo Task, namely attentional monitoring (e.g., of unexpected or infrequent stimuli) and updating of context information, inhibition of actions and interference, stimulus–response integration, and response selection.

The nCREANN analysis showed that there is a bi-directional transfer of information between all of these regions, underlining network accounts of inhibitory control^20^. Importantly, the correlation analysis of the network showed associations between directed connectivity parameters with individual sNFL concentrations. Since none of the individual sNFL values were in a range indicating pathological changes^62–65^, these results show that physiological variations in cytoarchitectonic integrity impact directed communication. Higher sNFL concentrations indicate a relatively weaker cytoarchitectonic integrity^34,36,99,100^. Since there were consistently positive correlations, higher sNFL levels were associated with stronger directed connectivity (information transfer) between the examined regions. Previous findings suggest that the organizational principles of theta network architecture become compromised with increasing sNFL concentrations^41^. The current findings show that, indeed, the directed information transfer between functional neuroanatomical structures is affected. This goes substantially beyond previous observations. The current finding of an increased directed information transfer between regions could be seen as a means to still enable sufficient information transfer when cytoarchitectonic integrity is affected. Interestingly, especially the degree of nonlinear directed information transfer is modulated. As shown in Fig. 3, nonlinear directed connections between all ACs involved were modulated by the sNFL level, while linear directed connections only revealed between-hemispheric correlations with the sNFL levels. To examine directed linear and nonlinear connectivity, nCREANN critically depends on the temporal profile of activity between brain regions to infer the temporal causality^61^. Thereby, nCREANN indirectly captures aspects of “signal fidelity” to infer directed communication. Signal fidelity refers to the accuracy and reliability with which a signal is transmitted from one point to another, maintaining the integrity of its original form. Signal fidelity is highly dependent on cytoarchitectonic integrity and white matter structure in particular^101–103^. While nCREANN models temporal activity patterns, it does not directly incorporate structural constraints, such as white matter integrity. Instead, the temporal precision of activity profiles indirectly reflects the influence of signal fidelity on directed communication. Moreover, in cases of disrupted structural connectivity, compensatory mechanisms (e.g., repeated signal transmission) may emerge to maintain effective communication^104,105^. Importantly, good signal fidelity is particularly crucial for functions that rely on precise timing, like sensory-motor integration, which is known to depend on the interplay of distributed processing in different brain regions^7,8^. Various studies revealed that tissue damage impacts nonlinear connectivity patterns in networks, such as the fronto-parietal control network (for review: ref. ^33^). Particularly, nonlinear functional connectivity is vulnerable to disruptions in the structural integrity of long-range white matter connections^33^. Therefore, it is reasonable that especially nonlinear directed was affected by weaker cytoarchitectonic integrity as indexed by sNFL. Interestingly, the data also show correlations between nonlinear information transfer and sNFL levels for the incompatible NOGO condition, i.e., where response inhibition was less demanding, but task set reconfiguration was more complex. To understand this, it is relevant that in incompatible trials, response selection cannot rely upon an ‘automated’ mapping between stimulus input (location) and the motor response. During incompatible trials in a Simon task, response selection (incl. the decision not to inhibit a response) requires a complex reconfiguration of the mapping of perceptual information on the motor output^78^, requiring network dynamics^106,107^.

In conclusion, this study reveals a relationship between neuronal cytoarchitectonic integrity and directed neurophysiological communication during cognitive control tasks. We demonstrated that higher sNFL concentrations, which indicate reduced cytoarchitectonic integrity, are associated with increased directed connectivity, particularly in nonlinear information transfer across the network, possibly to maintain sufficient communication between brain regions. The observed network-wide information exchange aligns with established accounts of inhibitory control involving distributed network interactions. These insights extend previous research by showing that physiological variations in neuronal integrity can significantly modulate functional network dynamics, thus influencing cognitive control mechanisms. Our findings provide a foundation for future investigations into the role of cytoarchitectonic integrity in other cognitive functions.

## Materials and methods

### Sample

In all, *N* = 55 healthy participants (35 female, mean age 33.8 ± 9.8 years, all right-handers) took part in the current study. Each individual had normal vision (or corrected to normal), normal hearing, and no history of mental or neurological disorders. All participants gave their informed, written consent. The TU Dresden Ethic Commission (EK 219062018) approved the study, and all procedures were conducted in accordance with the Helsinki Declaration, and all ethical regulations relevant to human research participants were followed. One participant had to be excluded from the final analysis due to missing sNFL values, leading to a final sample of *N* = 54 participants (34 female, mean age 33.6 ± 9.8 years). For behavioral results, the sample included *N* = 53 participants (33 female, mean age 33.3 ± 9.5 years), as the log file of the behavioral data (but not the EEG data) for one participant was missing.

### Task and procedure

**Fig. 5:**
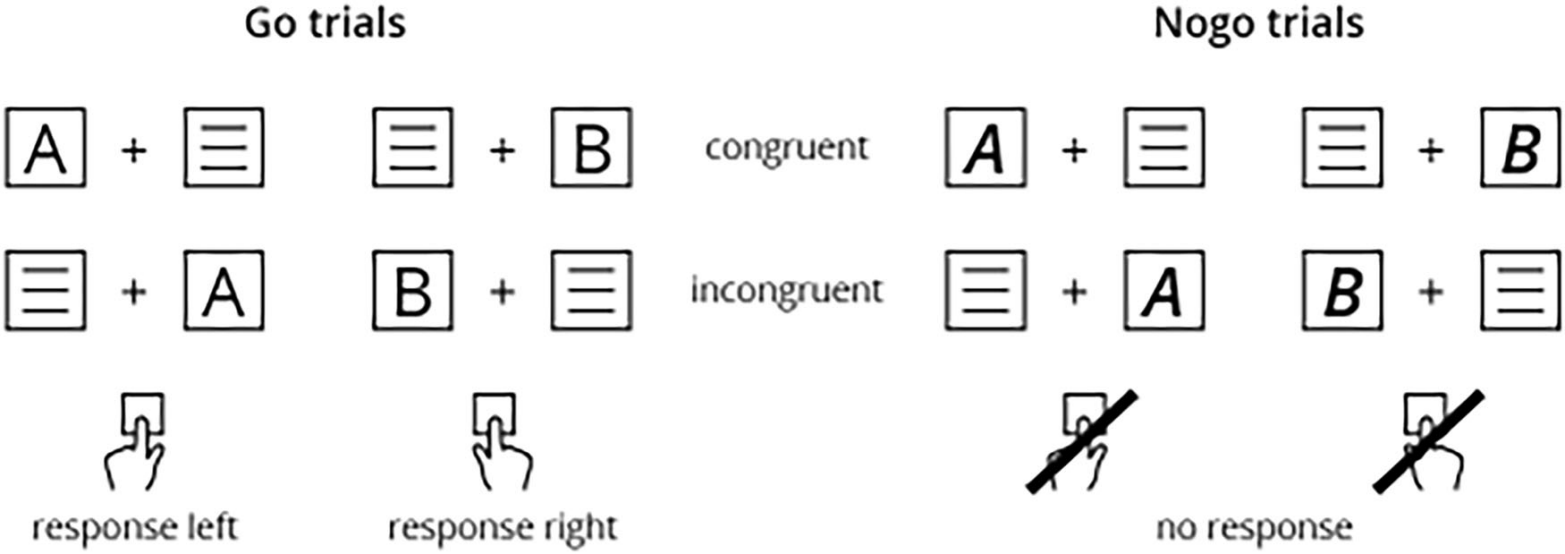
The Simon Nogo Task. Schematic illustration of possible stimuli combinations and corresponding responses in the Simon Nogo Task.

The Simon Nogo Task can be seen as a hybrid of a Simon Task and a Go/Nogo Task and allows to investigate the interplay between the “inhibition of interferences” and the “inhibition of actions”, specifically in the incompatible NOGO trials^4^. The procedure was as follows: participants should press the left “Ctrl” key when they saw the letter “A” and the right “Ctrl” key, when they saw the letter “B” on the screen, independent of its location (i.e., left or right). Thereby, the trials where the letter “A” appeared on the left side were compatible trials, while trials where “A” appeared on the right side were incompatible trials. For the letter “B” this pattern was vice versa. The previously explained procedure described a typical Simon Task. However, in the current study, we also implied a “NOGO” condition where participants should withhold their reaction (i.e., response inhibition). This was the case when letter “A” or “B” were written in bold-italic (see Fig. 5). Participants performed the experiment in a dimly lit room. They were seated 60 cm from a 24-inch display equipped with an eye-tracker. Prior to the main experiment, participants completed a 40-trial practice block for familiarization. The experiment consisted of 720 trials, divided into six blocks of 120 trials each, including 84 GO and 36 NOGO trials per block (i.e., 70:30 ratio). For both trial types, 50% were compatible and 50% incompatible, with stimuli and locations randomly assigned. Each trial began with a 200 ms stimulus, followed by a 1500 ms fixation. In GO trials, if no response occurred within 500 ms, a “Schneller” / “Faster” sign appeared to speed up reaction times. Trials ended either directly after a response was given or after 1700 ms, followed by a jittered inter-trial interval of 1100 to 1600 ms.

### EEG recording and preprocessing

EEG data was recorded with 60 spatial equally distributed Ag/AgCI electrodes using a QuickAmp or BrainAmp DC/ExG amplifier (Brain Products GmbH, Gilching, Germany), respectively. The sampling rate was 500 Hz. The recorded EEG signals were manually preprocessed in Brain Vision Analyzer (Brain Products GmbH, Gilching, Germany) with the following steps. First, the raw EEG signals were downsampled to 256 Hz and band-pass filtered for frequencies between 0.5 and 40 Hz. Artifacts were removed during a multi-step procedure. Technical or random artifacts (e.g., flat or bad channels, power spikes, or muscle tension) were removed manually, while periodically occurring artifacts (e.g., eye blinks, horizontal eye movements, and pulse artifacts) were removed using an Independent Component Analysis (ICA). If necessary, a second manual artifact rejection was applied. Afterward, all removed channels were back-interpolated. Following the EEG preprocessing, data were segmented in epochs locked on the stimulus onset (i.e., 2000 ms before and 2000 ms after of stimulus onset). As only the incompatible NOGO trials mirror the interplay between the “inhibition of interferences” and the “inhibition of actions”^4^, further EEG analysis steps were only conducted for the two NOGO conditions (i.e., compatible NOGO trials were used as comparison trials).

### Time-frequency decomposition and DICS beamformer

Further analysis of the data was conducted using Matlab and FieldTrip^108^. To reconstruct the source activity of the measured EEG signals for the theta frequency band, the DICS beamformer^109^, along with the forward model using the Montreal Neurological Institute (MNI) brain template^110^ were used. Using the Fieldtrip software, the MNI projects the localized brain activity from the DICS beamformer into a source space onto a grid with uniform spacing^108^. For the analysis of EEG data, this method has been shown to yield dependable results^69,111^. For the DICS analysis, we used a time window between 300 to 600 ms after the stimulus onset (i.e., cue-locked epochs). This time window was chosen based on the previous time-frequency analysis (see Fig. 2) and on previous literature on theta modulations in the Simon Nogo task^24,26^. We conducted a spectral analysis using a Hanning taper to calculate power and the cross-spectral density matrix for the theta frequency band. After aligning the EEG electrodes with the forward model, we created a leadfield matrix by dividing the brain volume into a 5 mm grid and calculating it for each grid point. A common spatial filter, with a 5% regularization parameter, was applied across both NOGO conditions. To correct for noise bias towards the center of the head, source estimation was normalized using a spatially inhomogeneous noise estimate. This noise estimate was based on the smallest eigenvalue of the cross-spectral density matrix^112^.

### Selection of regions of interest (ROIs) and LCMV beamformer

The regions of interest (ROIs) for the consecutive connectivity analysis were selected based on the results of the DICS beamformer. First, all voxels within 3% of the highest source activity values within functional neuroanatomical regions were selected. Next, these voxels were clustered using the DBSCAN algorithm^113,114^ with a minimum cluster size of three voxels. DBSCAN enables the detection of irregularly shaped clusters within distributed data points without requiring a predefined number of clusters. We selected an epsilon value of 1.5-times the grid size (i.e., the length of each voxel) to ensure that also voxels neighboring on an edge were detected. Based on the resulting clusters and associated AAL atlas labels^115^ ROIs were selected for each condition (i.e., compatible and incompatible NOGO trials). Virtual neural time series were computed for every voxel of each ROI and period using the Linearly Constrained Minimum Variance (LCMV) beamformer^112^. The time series for each voxel was computed by multiplying the LCMV spatial filter with the time-domain time series of the EEG data. Next, a band-pass filter (windowed sinc FIR filter) was applied on the resulting time series for the theta band frequency (4-7 Hz). The voltage values were averaged across voxels, separately for each condition and ROI.

### Directed connectivity analysis

Preprocessed EEG data was filtered for the theta frequency band using a Hamming windowed sinc FIR filter in order to prepare the data for the connectivity analysis, whereby the resulting time series had the same structure as the preprocessed EEG data. The time series was then segmented and categorized into two conditions separately. In the next step, the time courses of their underlying sources were extracted. To examine effective connectivity, we used a machine learning-based approach nCREANN^59–61^ to evaluate interactions between brain regions within the theta frequency band, focusing on ROIs derived from the DBSCAN algorithm in a previous step.

The nCREANN algorithm is based on a nonlinear Multivariate Autoregressive (nMVAR) model and employs an artificial neural network (ANN) to evaluate effective connectivity across brain regions. Unlike traditional linear approaches that rely solely on linear MVAR models, nCREANN captures both linear and nonlinear dynamics, which are essential for understanding the organization of information flow across cortical regions^116,117^. The nMVAR model reflects the nonlinear interactions between the past activity of the regions to generate their current states. This concept attempts to illustrate the temporal causality in which an effect influences the future. For a given time series ${{\bf{x}}}\left(n\right)\in {{\mathbb{R}}}^{M}$ of length L, a nonlinear MVAR model of order $p$ is defined as

2 $${{\bf{x}}}\left(n\right)={{\boldsymbol{f}}}({{{\bf{x}}}}_{p})+{{\boldsymbol{\sigma }}}(n)$$

Where ${{{\bf{x}}}}_{p}={\left[{x}_{1}\left(n-1\right),{x}_{2}\left(n-1\right),\cdots ,{x}_{M}\left(n-p\right)\right]}^{{{\rm{T}}}}$ is the vector of $p$ past samples of (M) multivariate time series. The noise vector, ${{\boldsymbol{\sigma }}}\left(n\right)=\quad {\left[{\sigma }_{1},{\sigma }_{2},\ldots ,{\sigma }_{M}\right]}^{T},$ is a real-valued zero-mean white noise vector. The nonlinear function ${{\boldsymbol{f}}}\left(.\right)$ quantitatively describes how the $p$ previous samples influence the future values. In the nCREANN method, the functions ${{\boldsymbol{f}}}$ is divided into linear and nonlinear part

3 $${{\boldsymbol{f}}}={{{\boldsymbol{f}}}}^{{Lin}}+{{{\boldsymbol{f}}}}^{{NonLin}}$$

Thus, based on the ${{{\boldsymbol{f}}}}^{{Lin}}$, the Linear Connectivity $({{lC}}_{i\to j})$ is calculated as the linear influence of $i$-th cluster on the $j$-th cluster, while based on the information embedded within ${{{\boldsymbol{f}}}}^{{NonLin}}$, the Nonlinear Connectivity $({{NC}}_{i\to j})$ is inferenced to establish the degree of the nonlinear causal influence of ${x}_{i}$ on ${x}_{j}$.

The time courses of the LCMV-derived sources in both compatible and incompatible NOGO conditions were subsequently analyzed with the nCREANN algorithm. For the connectivity analysis, the trial data points within the 300 to 600 ms time window were considered. To ensure a suitable length of data for training the network, all single-trial source signal values were concatenated. Using the Akaike and Schwartz criteria^118^, the ideal model order ($p$ = 7) was determined and was taken into account for every subject in both conditions. The significance of nCREANN outputs was evaluated using 100 surrogate datasets generated with a phase randomization algorithm. By dividing the connectivity values for each subject by the maximum value of both conditions for each linear and nonlinear connectivity measure, the values were normalized in a range between [0,1]. The connecting arrows are used to depict the connectivity patterns (see Fig. 3).

### Single-molecule array (SIMOA) analysis

sNFL samples were collected within a 7-day interval for behavioral and EEG measurements. After collection, serum samples were stored at −20 °C until sNFL measurements were conducted. sNFL concentrations were quantified using the Simoa Human Advantage NF-Light Singleplex Kit, following the manufacturer’s instructions (Quanterix, Lexington, MA; Datasheet: Simoa™ NF-Light® Advantage Kit). The Simoa Human Advantage NF-L assay, a digital immunoassay, was performed on the Simoa HD-X platform (Quanterix) using a 2-step Assay Neat 2.0 protocol^119^. The antibodies and calibrators, developed by Uman Diagnostics (Sweden), were originally described in the context of an ELISA-based method^120^ and adapted for the ultra-sensitive Simoa platform^121^. The Simoa NF-Light assay was validated as fit-for-purpose, with a lower limit of quantification of 0.0178 pg/ml (Datasheet Quanterix: Simoa™ NF-Light® Advantage Kit). Previous studies have demonstrated that the Simoa technology offers the highest sensitivity for detecting SNFLat very low concentrations^121^. Prior to analysis, a 96-well assay plate was prepared with calibrators, serum samples, and controls at room temperature (Quanterix). The assay components, including capture antibody-coated beads, a biotinylated detector antibody, streptavidin-beta-galactosidase, resorufin beta-D-galactopyranoside, and buffers, were loaded onto the Simoa HD-X Analyzer. The 2-step Neat protocol involved the simultaneous incubation of paramagnetic beads coated with target antibodies, the serum sample, and the biotinylated detector antibody. Target molecules in the sample were captured by the antibody-coated beads and bound with the biotinylated antibody detector simultaneously. Eight calibrator points, ranging from 0 to 500 pg/ml, and serum samples (diluted 1:4) were analyzed in duplicates, with dilutions calculated and performed by the instrument. Control measurements were predefined and ensured that concentrations remained within the expected range, validating the calibration curve and assay performance.

### Statistics and reproducibility

IBM SPSS Statistics 29.0.0.0 was used for the behavioral data analysis. We derived two factors from the Simon Nogo Task for the repeated measures ANOVA, namely *condition* (GO vs. NOGO) and *compatibility* (compatible vs. incompatible). Behavioral data were categorized into four conditions (i.e., Go compatible, Go incompatible, Nogo compatible, and Nogo incompatible). The Shapiro-Wilk Test showed violations of normal distribution for these four variables. However, since our sample was sufficiently large (*N* > 30)^122^ and an ANOVA with repeated measures is considered to be robust against the violation of the normal distribution assumption^123–126^, we did not apply a correction. A Greenhouse-Geisser correction was applied when necessary. We calculated averaged reaction times (RTs) and error rates for the compatible and incompatible GO trials as well as correct rejection rates for compatible and incompatible NOGO trials for each participant. Further, we used paired two-sided t-tests to compare performance between the conditions. Additionally, correlational analyses were conducted between behavioral results and sNFL data and between connectivity measures (i.e., nCREANN results) and sNFL data. The sample size is comparable to previous work using the same task and electrophysiological (i.e., EEG) methods^3,59^ and applying correlation analyses of sNFL and EEG data^41^. EEG data analyses used cluster-based permutation testing to control for type 1 errors (i.e., corrections for multiple comparisons). The analysis of network connectivity patterns is also presented using surrogate data. Correlational analyses were run between non-surrogated nCREANN data and sNFL values. The results were FDR-corrected for multiple comparisons.

### Reporting summary

Further information on research design is available in the  Nature Portfolio Reporting Summary linked to this article.

## Supplementary information

Reporting summary

## Data Availability

Raw data can be found in the Open Access Repository and Archive (OPARA) from TU Dresden 10.25532/OPARA-642^127^. The numerical source data for Fig. 1 can be found in logfiles and tables under the name “01_Behavioral data.zip”, numerical source data for Figs. 2, 3, and 4 can be found under the names “02_NFL data.zip” and “03_Preprocessed EEG data.zip”.

## Code availability

The custom code used to process the data can be found in OPARA 10.25532/OPARA-642^127^ under the name “04_Scripts.zip”.
