# Supplementary material for: Variations in neuronal cytoskeletal integrity affect directed communication in distributed networks during inhibitory control
Source: Commun Biol. 2025 Mar 29;8:516. doi: 10.1038/s42003-025-07974-4 (PMC11953345; doi:10.1038/s42003-025-07974-4)
Supplement: Supplementary file 1 — Reporting summary [file 42003_2025_7974_MOESM1_ESM.pdf]

nature portfolio

Corresponding author(s): Christian Beste

Last updated by author(s): Mar 12, 2025

Reporting Summary

Nature Portfolio wishes to improve the reproducibility of the work that we publish. This form provides structure for consistency and transparency in reporting. For further information on Nature Portfolio policies, see our Editorial Policies and the Editorial Policy Checklist.

Statistics

For all statistical analyses, confirm that the following items are present in the figure legend, table legend, main text, or Methods section.

n/a	Confirmed	
☐	☒	The exact sample size (*n*) for each experimental group/condition, given as a discrete number and unit of measurement
☐	☒	A statement on whether measurements were taken from distinct samples or whether the same sample was measured repeatedly
☐	☒	The statistical test(s) used AND whether they are one- or two-sided<br>*Only common tests should be described solely by name; describe more complex techniques in the Methods section.*
☒	☐	A description of all covariates tested
☐	☒	A description of any assumptions or corrections, such as tests of normality and adjustment for multiple comparisons
☐	☒	A full description of the statistical parameters including central tendency (e.g. means) or other basic estimates (e.g. regression coefficient) AND variation (e.g. standard deviation) or associated estimates of uncertainty (e.g. confidence intervals)
☐	☒	For null hypothesis testing, the test statistic (e.g. *F*, *t*, *r*) with confidence intervals, effect sizes, degrees of freedom and *P* value noted<br>*Give P values as exact values whenever suitable.*
☒	☐	For Bayesian analysis, information on the choice of priors and Markov chain Monte Carlo settings
☒	☐	For hierarchical and complex designs, identification of the appropriate level for tests and full reporting of outcomes
☐	☒	Estimates of effect sizes (e.g. Cohen's *d*, Pearson's *r*), indicating how they were calculated

Our web collection on statistics for biologists contains articles on many of the points above.

Software and code

Policy information about availability of computer code

Data collection	The behavioral experiment was programmed with Presentation® (Neurobehavioral Systems). EEG data was recorded with BrainVision Recorder (Brain Products GmbH, Gilching, Germany).
Data analysis	Behavioral data was analyzed with IBM SPSS Statistics 29.0.0.0 and MATLAB 2020b. The recorded EEG signals were manually preprocessed in Brain Vision Analyzer (Brain Products GmbH, Gilching, Germany) and further analyzed with the FieldTrip Toolbox in MATLAB 2020b (The MathWorks). NFL concentrations were quantified using the Simoa Human Advantage NF-Light Singleplex Kit, following the manufacturer's instructions (Quanterix, Lexington, MA; Datasheet: Simoa™ NF-Light® Advantage Kit).

For manuscripts utilizing custom algorithms or software that are central to the research but not yet described in published literature, software must be made available to editors and reviewers. We strongly encourage code deposition in a community repository (e.g. GitHub). See the Nature Portfolio guidelines for submitting code & software for further information.

Data

Policy information about availability of data

All manuscripts must include a data availability statement. This statement should provide the following information, where applicable:

- Accession codes, unique identifiers, or web links for publicly available datasets
- A description of any restrictions on data availability
- For clinical datasets or third party data, please ensure that the statement adheres to our policy

Data Availability Statement
The numerical source data for Figures 1, 2, 3, and 4 can be found in the tables and scripts that have been provided in the Open Access Repository and Archive (OPARA) from TU Dresden.
Raw data can be found here: https://doi.org/10.25532/OPARA-642

Code Availability Statement
Custom code used to process the data can be found here: https://doi.org/10.25532/OPARA-642

Research involving human participants, their data, or biological material

Policy information about studies with human participants or human data. See also policy information about sex, gender (identity/presentation), and sexual orientation and race, ethnicity and racism.

Reporting on sex and gender	Gender was not considered in this study.
Reporting on race, ethnicity, or other socially relevant groupings	No socially constructed categorizations were used.
Population characteristics	see below
Recruitment	Participants were recruited using an internal database for study participants in child and adolescent psychiatry at Dresden University Hospital. Participants had indicated in previous studies that they may be contacted for further studies.
Ethics oversight	The TU Dresden Ethic Commission (EK 219062018) approved the study and all procedures were conducted in accordance with the Helsinki Declaration and all ethical regulations relevant to human research participants were followed.

Note that full information on the approval of the study protocol must also be provided in the manuscript.

Field-specific reporting

Please select the one below that is the best fit for your research. If you are not sure, read the appropriate sections before making your selection.

☐ Life sciences ☒ Behavioural & social sciences ☐ Ecological, evolutionary & environmental sciences

For a reference copy of the document with all sections, see nature.com/documents/nr-reporting-summary-flat.pdf

Behavioural & social sciences study design

All studies must disclose on these points even when the disclosure is negative.

Study description	In this cross-sectional study quantitave data (EEG, behavioral, blood) was collected.
Research sample	N = 55 healthy participants (35 female, mean age 33.8 ± 9.8 years, all right-handers)
Sampling strategy	The sample size was chosen based on comparable previous work using the same task and electrophysiological (i.e., EEG) methods (Chmielewski et al., 2018; Elmers et al., 2024) and applying correlation analyses of sNFL and EEG data (Beste et al., 2019).
Data collection	Participants performed the Simon Nogo Task alone in a dimly lit room, while EEG data was recorded with 60 spatial equally distributed Ag/AgCl electrodes using a QuickAmp or BrainAmp DC/ExG amplifier (Brain Products GmbH, Gilching, Germany). Blood samples were drawn by trained professionals at the MS Center Dresden within 7 days prior or post to the EEG experiment.
Timing	First data set was collected in December 2019 and last data set was collected in August 2023. Data collection was ongoing, but slowed in the years 2020 and 2021 due to regulations adressing the Covid pandemic.
Data exclusions	One participant was excluded due to missing NFL values from the whole analysis; One participant was excluded from behavioral analysis due missing logfile of the behavioural data (but not the EEG data).

Non-participation	No participant dropped out during the study.
Randomization	n/a

Reporting for specific materials, systems and methods

We require information from authors about some types of materials, experimental systems and methods used in many studies. Here, indicate whether each material, system or method listed is relevant to your study. If you are not sure if a list item applies to your research, read the appropriate section before selecting a response.

Materials & experimental systems

n/a	Involved in the study
☒	☐ Antibodies
☒	☐ Eukaryotic cell lines
☒	☐ Palaeontology and archaeology
☒	☐ Animals and other organisms
☒	☐ Clinical data
☒	☐ Dual use research of concern
☒	☐ Plants

Methods

n/a	Involved in the study
☒	☐ ChIP-seq
☒	☐ Flow cytometry
☒	☐ MRI-based neuroimaging

Plants

Seed stocks	n/a
Novel plant genotypes	n/a
Authentication	n/a
